# Motion Discrimination and the Motion Aftereffect in Mouse Vision

**DOI:** 10.1523/ENEURO.0065-18.2018

**Published:** 2018-12-21

**Authors:** Jason M. Samonds, Sarina Lieberman, Nicholas J. Priebe

**Affiliations:** 1 Department of Neuroscience; 2 Center for Perceptual Systems; 3 Center for Learning and Memory, University of Texas at Austin, Austin, Texas 78712

**Keywords:** classical conditioning, motion aftereffect, motion coherence, mouse, vision

## Abstract

Prolonged exposure to motion in one direction often leads to the illusion of motion in the opposite direction for stationary objects. This motion aftereffect likely arises across several visual areas from adaptive changes in the balance of activity and competitive interactions. We examined whether or not the mouse was susceptible to this same illusion to determine whether it would be a suitable model for learning about the neural representation of the motion aftereffect. Under a classical conditioning paradigm, mice learned to lick when presented with motion in one direction and not the opposite direction. When the mice were adapted to motion preceding this test, their lick behavior for zero coherence motion was biased for motion in the opposite direction of the adapting stimulus. Overall, lick count versus motion coherence shifted in the opposite direction of the adapting stimulus. This suggests that although the mouse has a simpler visual system compared with primates, it still is subject to the motion aftereffect and may elucidate the underlying circuitry.

## Significance Statement

The motion aftereffect is a well-known illusion that provides insight about motion processing and adaptation throughout the visual system. We demonstrate that motion discrimination in mice is affected by extended viewing of coherent motion, in a manner consistent with the effects reported in primates. The mouse model currently offers unprecedented experimental tools for studying neural circuitry and our results support the mouse as a model to study this phenomenon.

## Introduction

Prolonged exposure to sensory stimuli induces systematic changes in the way that we perceive the world. One such change in our perception is the “waterfall” illusion, in which we perceive motion of stationary objects right after observing moving objects for an extended period. This perceived motion is in the opposite direction of the moving objects. Observing the downward movement of a waterfall causes surrounding stationary objects to appear as if they are moving upward (Addams, 1834). The neural basis of this motion aftereffect has been elusive but is thought to reflect imbalance and competition in the responses of motion-selective neurons in multiple visual areas (Anstis et al., 1998).

To explore the neural circuitry underlying the motion aftereffect, we sought to develop a motion discrimination paradigm in mice, because there is an array of transgenic mice that would allow us to manipulate and measure individual circuit elements. Early attempts to train mice to perform motion discrimination of opposite directions were not successful (Douglas et al., 2006) though, suggesting that perhaps motion information is processed in a distinct manner from other mammals. One striking difference between mice and primates is the presence of a strong functional organization in the visual cortex: rodents lack a functional organization beyond retinotopy, whereas primates and carnivores have a columnar organization for orientation, direction, and spatial frequency (Hubel and Wiesel, 1962, 1968; Malonek et al., 1994; Girman et al., 1999; Issa et al., 2000; Ohki et al., 2005; Nauhaus et al., 2012). Although it is tempting to link this difference in the organization of sensory information across species to changes in motion processing, rats, which share the lack a functional organization in visual cortex, are able to perform motion discrimination tasks (Douglas et al., 2006). Additionally, a more recent study has demonstrated that mice are able to discriminate motion in opposite directions using a different training paradigm (Marques et al., 2018).

We set out to determine whether mice could discriminate motion using classical conditioning and to see whether their report of motion depends on the recent history of motion stimulation. We demonstrate here that mice can accurately discriminate motion of opposite directions and that their report is subject to the waterfall illusion: e.g., prolonged visual stimulation with rightward motion induces mice to report that leftward motion exists for conditions in which no coherent motion is presented. These results demonstrate that motion processing in the mouse visual system, although differing in functional organization from primates, exhibit similar integrative and competitive processes, which result in the illusion of motion to stationary stimuli following prolonged stimulation.

## Materials and Methods

### Preparation of animals

Two adult female C57BL/6 and two adult PV-Cre;ChR2 (1 male, 1 female) on a C57BL/6 background mice (4-18 months) were used in these experiments. To immobilize the head during behavior, a titanium bar was placed on the skull and secured with dental acrylic under isoflurane (1-3%) anesthesia (Samonds et al., 2018). All procedures were approved by the Institutional Animal Care and Use Committee and conformed to National Institutes of Health standards.

### Visual stimuli

We used a DepthQ HDs3D2 projector (DepthQ/Lightspeed Design) with a refresh rate of 120 Hz at full HD resolution (1920 × 1080), operating in gray-scale mode (mean luminance = 59.75 cd/m^2^). Stimuli were either rear-projected onto a polarization-preserving screen (Da-Lite 3D virtual black rear screen fabric, model #35929) or front-projected onto a silver polarization-preserving screen (Severtson, SeVision 3D GX, 2.2 Silver). One pixel subtended 0.1° at a viewing distance of 22 cm.

Black and white dot motion stimuli of varying coherence were generated using MATLAB (MathWorks) and Psychtoolbox (Brainard, 1997). Each of 800 dots of 1° of visual field in diameter were displayed within a 60° square aperture in front of the mouse, appeared in random locations, and moved in a given direction for a dot lifetime of six frames. The dot lifetime restricted the highest possible coherence to 86% (Bischof et al., 1999). Each trial consisted of a 3 s visual stimulation period and were separated by an interstimulus period lasting 10 s plus a random interval drawn from an exponential distribution with a mean of 10 s. This random interval prevents the mice from predicting the time point of reward delivery (Khastkhodaei et al., 2016; Jurjut et al., 2017). During preliminary motion discrimination training, we displayed dots with 86% coherent motion and 100% Michelson contrast during the stimulation period, and we presented a mean gray screen during the interstimulus period. During motion aftereffect tests, we presented dots of varying coherent motion ranging from 0 to 51% during 4 s of stimulation with 100% contrast, and we presented 0% coherent motion dots, 86% coherent leftward moving dots, or 86% coherent rightward moving dots at 30% contrast during the randomly timed interstimulus period. There was a mean gray screen gap of 0.5 s between the adaptation stimulus and the test stimulus conditions, and a gap of 5 s following stimulation, in which a water reward was delivered.

### Training paradigm

All animals were water-restricted for 1 week before training and the weights of the animals were maintained within 30% of the original body weight (Guo et al., 2014). The animals were acclimated to the training apparatus during the restriction period. Animals walked on a floating Styrofoam ball while they were head-fixed (Dombeck et al., 2007). Classical conditioning was used to pair visual motion to water delivery (Khastkhodaei et al., 2016; Jurjut et al., 2017). Initially, we presented two different motion conditions, upward and leftward, pairing leftward with a water reward. Over a few weeks of training sessions, mice associated the reward with leftward motion and began to lick before the water being delivered at the end of stimulation and only for the leftward moving stimuli. Once trained to associate water with leftward motion, the mice quickly adapted (typically less than a few days) to small changes in the experimental paradigm (e.g., changes in the direction of unrewarded stimuli, coherence, adapting experiments). Licking was measured using a piezoelectric sensor attached to the reward delivery system (Schwarz et al., 2010; Khastkhodaei et al., 2016; Jurjut et al., 2017). The water was delivered in a flexible catheter that would bend upward and downward as the mouse licked. All behavior was quantified as single lick events and lick rates that were detected using a threshold based on the typical output of the piezoelectric sensor. For each stage of training, we gradually exposed the mice to any particular change (direction of motion, coherence, adapter) sometimes combining the changes with the original conditions until they were conditioned to the test stimuli.

### Eye tracking

During all of the experimental procedures and training, an IR camera recorded video of the eye movements at 30 Hz. An artificial eye with a diameter of 3.1 mm was used to calibrate eye position estimates. Eye positions were extracted (based on the center of the pupil) and analyzed using custom MATLAB software (Samonds et al., 2018). We did not find any changes in eye position or velocity that correlated with the direction of motion of presented stimuli because we used a motion speed (30 degrees/s) outside of the range of measurable ocular kinetic reflex gains in mice (Tabata et al., 2010).

### Statistical analysis

Using the least-squares curve fit function in MATLAB, we fit the following sigmoid function for all mean lick counts (*L*) versus coherence (*c*) data:

(1) $$L=\left(M-u\right)\frac{1}{1+e^{-b\left(c-c_{o}\right)}}+u,$$

where *M* and *u* determine the vertical position of the sigmoid (maximum and minimum lick counts), *b* determines the slope of lick counts with coherence, and *c_0_* determines lateral position (bias) of the sigmoid (coherence). This function was also used to fit a function for *d*′ versus coherence and an inverted sigmoid function was used to fit a function for latency versus coherence. Confidence intervals (CIs) and significance were estimated using a bootstrap procedure. We resampled our datasets randomly with replacement 1000 times to generate bootstrap distributions. The 68% CIs (SE) were defined as the resampled data corresponding to the (16, 84) percentile range of the bootstrap distributions.

## Results

Here we examined whether sensory adaptation alters the perception of motion in the mouse. We initiated our experiments by first training animals to discriminate leftward and upward motion using classical conditioning (Khastkhodaei et al., 2016; Jurjut et al., 2017) and then slowly rotated the upward motion to the right until the task was a leftward and rightward motion discrimination. Once mice reached a criterion performance (*d*′ ≥ 0.8) in this task, we measured how the history of sensory stimulation impacted their ability to discriminate these directions.

### Training mice to discriminate opposite directions

**Figure 1.**
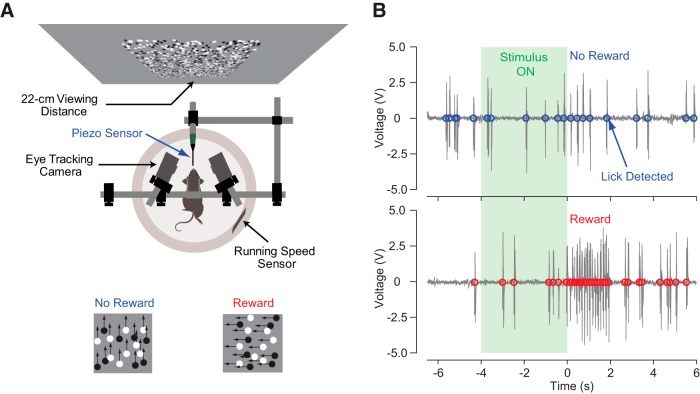
Experimental setup. ***A***, Random-dot motion stimuli were projected in front of mice running on a floating track ball. A water reward was given to mice following the presentation of only motion in the leftward direction. ***B***, Licks were measured using a piezoelectric sensor attached to the water delivery system.

Previous studies have reported that it is difficult to train mice to perform direction discrimination tasks when the motion directions are opposing whereas they can perform tasks when the directions are orthogonal (Douglas et al., 2006; Stirman et al., 2016). With these studies in mind, we first paired a water reward with leftward motion and presented an upward motion stimulus without any reward. We placed head-fixed mice on a ball floating on air (Fig. 1*A*) and measured the licking behavior of animals using a piezoelectric sensor placed on a waterspout (Fig. 1*B*).

**Figure 2.**
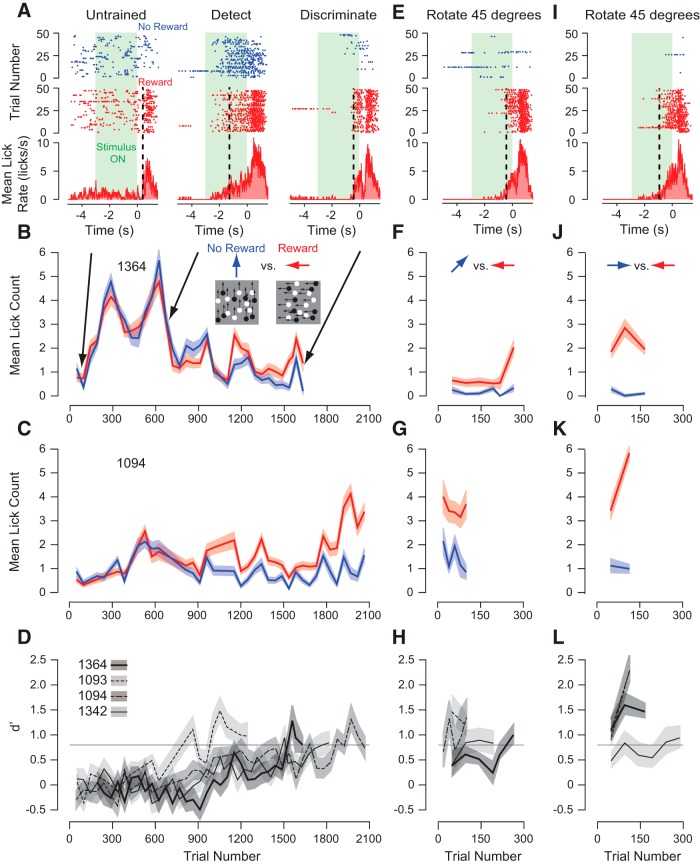
Classical conditioning paradigm for learning to discriminate the direction of motion. ***A***, Example lick raster plots and histograms for 3 sessions for one of the mice (1364). Initially (Untrained), this mouse licked spontaneously and infrequently before and rigorously after the reward was delivered (left). After several hundred trials, this mouse began licking more frequently in anticipation of a water reward for both moving stimuli (center, Detect). Finally after 1500 trials, the mouse licked in anticipation for only the rewarded leftward motion stimulus (right, Discriminate). Vertical dashed line is the lick onset latency. ***B***, Mean lick count from −1 to 0.2 s for rewarded (red) and unrewarded (blue) directions of motion over all initial sessions of training for the data shown in ***A***, which is a mouse that decreased licking to the unrewarded condition (blue). ***C***, Mean lick count for a mouse that increased licking to the rewarded condition (red). ***D***, *d*′ Calculated for each session from the lick counts shown in ***B*** and ***C***, as well as from lick counts measured from the other two mice. Horizontal dashed line is the training criterion. ***E***, After the mice learned to discriminate upward from leftward motion, we slowly rotated the unrewarded stimulus. An example lick raster plot and histogram is shown for Mouse 1364. ***F*–*H***, There was still a difference in lick count and significant *d*′, and these values even continued to increase when rotating the unrewarded stimulus by 45°. ***I***, After the mice learned to discriminate oblique from leftward motion, we rotated the unrewarded stimulus another 45° so the unrewarded condition was rightward motion. Again, an example lick raster plot and histogram is shown for Mouse 1364. ***J*–*L***, Again, there was still a difference in lick count and significant *d*′, and discrimination even continued to improve for leftward versus rightward motion. All error bars are bootstrapped SEM and *d*′.

Motion stimuli were presented for 3 s, and initially animals licked randomly and only vigorously once water came out of the spout (Fig. 2*A*, Untrained). For all naive mice, lick rate increases occurred 400 ms after the water exited the spout (Untrained phase latency range: +402 to +445 ms; Fig. 2*A*, dashed black line). Latency for licking was defined as the time to reach 20% of the peak lick rate relative to reward onset. Each day, we trained mice using 96 trials, 48 rewarded leftward and 48 unrewarded upward motion conditions. Within 1 week, the mice started to associate the stimulus with the reward, but initially without distinguishing between the rewarded and unrewarded direction of motion. The mice reduced their spontaneous licking and started to lick soon after the motion stimulus turned on and more vigorously after the reward was delivered for the rewarded condition (Fig. 2*A*, Detect). The mice anticipated a reward once detecting a motion stimulus by starting to lick over a second (Detection phase latency range: −1394 to −2636 ms) before the reward was delivered (Fig. 2*A*, Detect, black dashed line). After a couple more weeks of conditioning, the mice began to associate only the leftward motion stimulus with the water reward (Fig. 2*A*, Discriminate). Mice continued to lick before the appearance of the water stimulus (Fig. 2*A*, Discriminate, black dashed line), but did not start licking as quickly as during the detection phase (Discrimination phase latency range: −385 to −633 ms).

Another effect of training was that the lick rate difference between the rewarded and unrewarded stimulus progressively increased. We measured lick count during the period between −1 and +0.2 s relative to reward onset. This window was chosen based on: (1) the latency of discriminatory licking relative to reward onset being <1 s, (2) the latency of licking relative to reward onset for unconditioned mice being >400 ms, and (3) a bifurcation observed in lick rate around 400 ms after reward onset suggesting a distinction between licking based on the stimulus and licking based on the delivery of the water (Fig. 2*A*,*E*,*I*). As training progressed over 3-4 weeks, mice decreased their lick count to the unrewarded direction of motion (Fig. 2*B*, blue) and/or increased their lick count for the rewarded direction of motion (Fig. 2*C*, red). To quantify the degree to which lick rate reflected a discrimination between leftward and upward motion we computed *d*′ between lick counts on a day-by-day basis (Fig. 2*D*). Over the conditioning period, *d*′ slowly increased reaching values consistently >0.8, which was our criterion to begin the shaping process.

**Figure 3.**
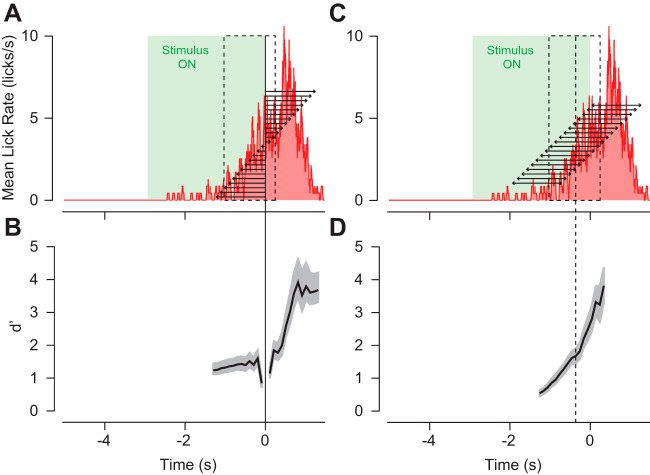
Dependence of discrimination of lick count window. ***A***, The lick rate over time for the rewarded condition increases before the end of the stimulus presentation (green), and continues increasing at an even greater rate 400 ms after the reward is delivered. We measured *d*′ in progressively larger windows (arrows) on both side of reward onset. The window used in Figure 2 is shown as a dashed black box. ***B***, The resulting *d*′ measurements between the rewarded and unrewarded conditions for the increasing window sizes described in ***A***. ***C***, We also measured *d*′ while moving a window with a fixed size of 1.2 s from −800 ms before to +800 ms after reward onset. ***D***, The resulting *d*′ measurements for the window positions described in ***C***. The vertical dashed line is the center of the window used in Figure 2. Shaded regions are the bootstrapped SE of *d*′.

Our analysis of behavioral performance does depend on the timing over which we measured lick rate. To examine how our analysis window affected our discrimination measurements we measured *d*′ for the licking behavior at the end of this training period (Figs. 2*I*, 3*A*) with progressively larger windows starting from each side of reward onset. We find that there are minor changes in *d*′ as we increase the duration of the pre-reward window size (Fig. 3*A*, leftward arrows), with a slight decrease in *d*′ as we include longer pre-reward intervals (Fig. 3*B*, left). Including increasing amounts of post-reward intervals (Fig. 3*A*, rightward arrows) has little initial influence on *d*′ until 400 ms, at which time *d*′ increases dramatically (Fig. 3*B*, right). At that time, the mouse was responding to reward delivery by licking vigorously rather than anticipating a reward because of the stimulus. We also examined how shifting a window with a fixed size of 1.2 s varied *d*′ estimates (Fig. 3*C*). As we moved the window progressively closer to reward onset, *d*′ increased at a constant rate. As the leading (latest) edge of the window approached 100 ms after reward onset, *d*′ changed minimally (Fig. 3*D*, center of window at 500 ms before reward onset: vertical dashed line). When the leading edge of the window reached 400 ms after reward onset, *d*′ increased at a rate more rapidly than before reward onset because the mouse was responding to reward delivery rather than licking in anticipation of a reward. Both analyses support our choice of the interval of −1 to +0.2 s (Fig. 3*A*, black dashed box) to measure lick behavior, which includes the majority of the anticipatory licking (Fig. 3*A*, red) that was induced by our conditioning paradigm.

For this study, we were primarily interested in how the history of sensory information influences motion perception. For example, in the waterfall illusion, prolonged downward motion elicits a perception of upward motion, but may not alter perceptions of rightward and leftward motion. Although our classical conditioning paradigm demonstrates that mice can successfully discriminate leftward and upward motion as previously reported (Douglas et al., 2006; Stirman et al., 2016), we needed to observe mice discriminating leftward and rightward motion to induce a motion aftereffect. To change our task to one in which mice discriminate motion along the horizontal axis, we used a paradigm over a series of days in which we rotated the upward moving stimulus toward the rightward direction. Initially, we rotated the stimulus at 45° and mice could still discriminate the leftward and oblique directions (Fig. 2*E*–*H*). We continued rotating the stimulus until the mice could distinguish leftward and rightward directions of motion (Fig. 2*I*–*L*). The mice rapidly generalized their discriminatory lick behavior to the change in direction of motion for the unrewarded condition (Fig. 2*H*,*L*) and even continued to improve their discrimination over these periods (Fig. 2*L*). For the last data point in Figure 2*L*, we included *d*′ measurements from lick behavior following all the subsequent stages of training described below to illustrate that the mice retained their discriminability or even had improved performance although the task continued to become more difficult because we reduced motion coherence (described in the next section).

### Using the motion aftereffect to bias motion discrimination

**Figure 4.**
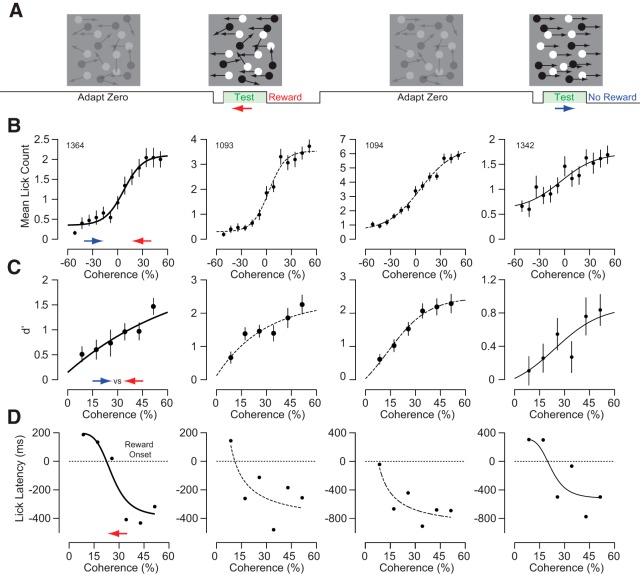
Motion discrimination improves with increasing random dot motion coherence. ***A***, Mice were shown a 30% contrast zero coherence adapting stimulus for at least 10 s before being shown a 4 s 100% contrast test stimulus of varying coherence. ***B***, Lick count increased monotonically as a sigmoid function versus coherence for all of the mice (solid and dashed black lines). Error bars are bootstrapped SEM (*n* = 62–70 repeats for each condition). ***C***, *d*′ also increased monotonically as a sigmoid function versus coherence for all of the mice (solid and dashed black lines). Error bars are bootstrapped SE of *d*′. ***D***, The mice all licked sooner for higher coherence leftward motion as a sigmoid function of coherence as well.

Once mice could discriminate leftward and rightward moving stimuli, we measured the impact of long-term motion adaptation and changing the strength of motion on behavior. To examine how the history of motion stimulation impacts the discriminability behavior of the mice, we varied the coherence and direction of dots during an adaptation phase and during the test phase. The adapting phase occurred during the interstimulus period of the conditioning paradigm (Fig. 4*A*) and was composed of noncoherent motion or full coherent motion in the rightward or leftward directions at low contrast. The test phase was composed of moving dots of systematically different coherence with high contrast (Douglas et al., 2006; Stirman et al., 2016; Marques et al., 2018). Coherence is defined as the percentage of dots moving in the same leftward or rightward direction. The rest of the dots moved in random directions.

Licking behavior was systematically related to test coherence when the adapting motion contained 0% coherence dots (Fig. 4*B*–*D*). As test coherence for leftward motion increased, lick count increased until saturating at a maximum number of licks (Fig. 4*B*, right side of each plot). As test coherence for rightward motion increased, lick count decreased until saturating near zero licks (Fig. 4*B*, left side of each plot). Although maximum mean anticipatory lick counts varied among mice (2–6 licks), their discriminability was similar. We measured *d*′ for lick counts for each mouse to stimuli of equal coherence, but opposite direction (Fig. 4*C*). As expected based on lick counts, *d*′ increased with increasing test coherence maxing out at *d*′ = 1–2.5. Additionally, the mice generally started to lick earlier for higher compared to lower coherence leftward motion stimuli. The corresponding latencies for lick behavior to leftward motion stimuli in Figure 4*B* are shown in 4*D*. All of the mice started licking sooner for leftward motion with higher coherence.

**Figure 5.**
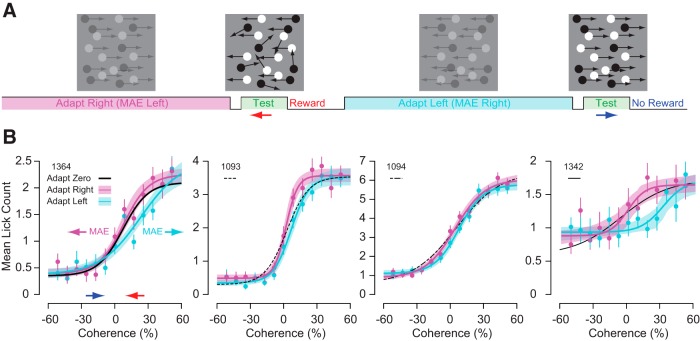
Motion discrimination is biased by previous prolonged exposure to motion stimuli. ***A***, Mice were shown a 30% contrast fully coherent adapting stimulus for at least 10 s before being shown a 4 s 100% contrast test stimulus of varying coherence. ***B***, Mean lick counts for rightward (magenta) and leftward (cyan) adapting conditions. The corresponding sigmoid fits from Figure 4*B* for zero coherence adapted lick counts versus coherence are displayed as solid and dashed black lines. The mice licked more for rightward adapting stimuli (magenta) because this should produce a leftward motion aftereffect, which corresponds to the rewarded condition. The mice licked less for leftward adapting stimuli (cyan) because this should produce a rightward motion aftereffect, which corresponds to the unrewarded condition. Error bars are SE and shaded bands for magenta and cyan lines are the bootstrapped SE of sigmoid fits (*n* = 62–70 repeats for each condition).

**Figure 6.**
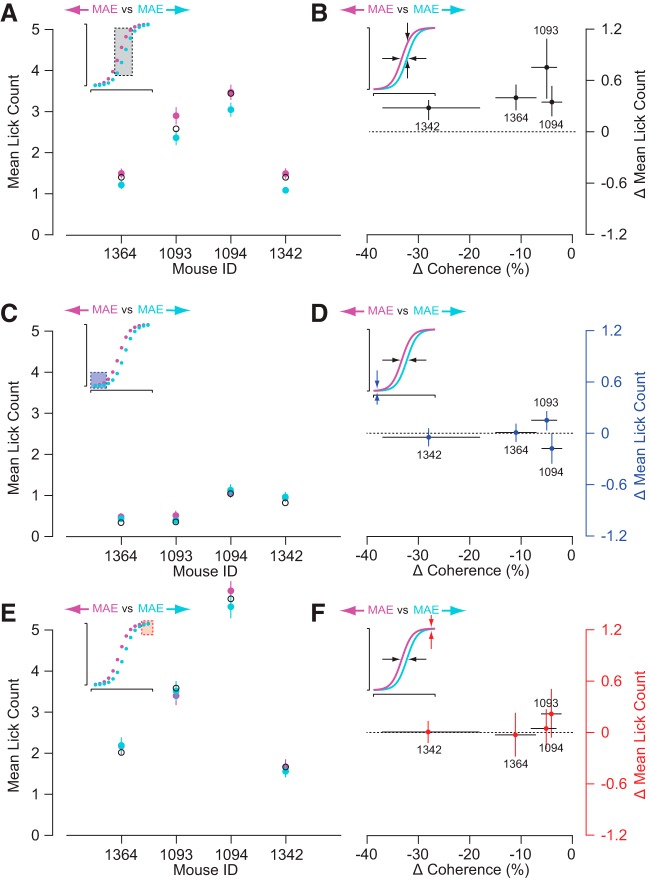
Licking behavior was consistent with a motion aftereffect. ***A***, Average lick counts for the four coherences along the steepest slope in Figure 5*B* for the rightward (magenta) and leftward (cyan) adapting conditions (inset, gray box). For all four mice, the rightward-adapted conditions had significantly more licking than the leftward-adapted conditions (*p* < 0.01). ***B***, Measurements of the shifts in the coherence bias and mean lick rate following rightward and leftward adaptation, based on sigmoid fits (inset). The dashed line indicates no change in lick rate. All data points represent upward and leftward shifts between the functions for the four mice and were significantly above (*y*-axis) and below (*x*-axis) zero, respectively (*p* < 0.05). ***C***, Average lick counts for the three highest coherences for rightward motion in Figure 5*B* for the rightward- (magenta) and leftward- (cyan) adapted conditions (inset, blue box). For all four mice, there was no significant difference between the rightward- and leftward-adapted conditions (*p* > 0.05). ***D***, As in ***B***, but the ordinate plots differences in the lower asymptotes of the sigmoid function. For all four mice, there was also no significant difference in the lower asymptotes of the sigmoid function (inset) between the leftward-adapted function (magenta) compared with the rightward-adapted function (cyan; *p* > 0.05). ***E***, Average lick counts for the two highest coherences for leftward motion in Figure 5*B* for the rightward- (magenta) and leftward- (cyan) adapted conditions (inset, red box) were also not significantly different (*p* > 0.05). ***F***, As in ***B*** and ***D***, but the ordinate plots the differences in the upper asymptotes. There was no significant difference in the upper asymptotes of the sigmoid function (inset) between the leftward-adapted function (magenta) compared with the rightward-adapted functions (cyan; *p* > 0.05). ***A***, ***C***, and ***E***, Error bars are bootstrapped SE of the average and open black circles are the lick counts for the 0% coherence adapting condition (*n* = 62–70 repeats for each condition). ***B***, ***D***, and ***F***, Error bars are bootstrapped SE of sigmoid fit parameters.

To examine whether preceding motion altered their behavior, we measured responses to the test stimulus when there was full coherence motion in the leftward or rightward direction during the adapting phase (Fig. 5*A*). As with the previous experiments, the mouse was then rewarded following only leftward moving test stimuli. The adapting motion had systematic effects on the licking behavior of the mice in response to the test stimulus. In humans, exposure to rightward motion causes the misperception of leftward motion to stationary stimuli and exposure to leftward motion causes the misperception of rightward motion to stationary stimuli. Based on psychometric functions observed for human motion aftereffect studies, we predicted that coherent motion during the adapting phase would shift the lick count versus coherence functions (Fig. 4*B*) to the left or right if the motion of the adapter is rightward or leftward, respectively (Blake and Hiris, 1993; Van Wezel and Britten, 2002; Czuba et al., 2011). Consistent with the motion aftereffect illusion, mice licked more in response to stimuli at zero coherence following rightward adaptation than leftward adaptation (Fig. 5*B*, magenta vs cyan traces). We quantified differences in lick rates between the rightward (magenta) and leftward (cyan) adapting conditions by comparing the lick counts for the four coherences along the steepest slope of the response curves in Figure 5*B*, where the mice had the most uncertainty (e.g., 0–25.7%; Fig. 6*A*, inset). Within this range for all of the mice, there was significantly more licking for the rightward adapting condition versus the leftward adapting condition (Fig. 6*A*, magenta vs cyan data points; *p* < 0.01, bootstrapping). To further evaluate our prediction, we measured whether adaptation altered lick behavior by fitting a sigmoid function to the data and (1) computing the mean difference in coherence at the halfway point between the minimum and maximum lick counts of the sigmoid fits and (2) calculating the mean lick count difference of the fits at the halfway point of the leftward adapting sigmoid fit (Fig. 6*B*). Both characterizations (horizontally and vertically) of the sigmoid shift are statistically significant (*p* < 0.05) for all mice based on bootstrap analysis. For some of the mice, the zero coherence adapted sigmoid function is shifted to the right relative to zero coherence (Fig. 5*B*, Mouse 1364). This is likely because these mice only licked when there was leftward motion detected. Some mice licked less when there was high coherence for rightward motion and licked more when there was high coherence for leftward motion (Fig. 5*B*, Mouse 1094). Despite these intersubject differences, we still observe consistent shifts in lick behavior that depend on the adaptation direction. Overall, our analysis supports that the adapter shifted the response curves in the expected directions if there were a motion aftereffect for mice.

We designed our motion aftereffect paradigm so that the mice would ignore the adapting stimulus by lowering the contrast relative to the test stimulus (Fig 5*A*). Nonetheless, the adapter stimuli could have changed motivation or arousal systematically that would produce a difference between the rightward and leftward lick counts consistent with Figure 6, *A* and *B*. For example, if the mice associated rightward motion during the adapting phase with increased reward, they might lick more for all test conditions or they might have increased sensitivity for all leftward test conditions. This behavior would produce an upward shift, or multiplicative increase, in the licking behavior with coherence.

To determine whether the adapting stimuli caused behavioral changes such as increased or decreased motivation or arousal, we tested for differences in lick counts at high coherences for both rightward and leftward motion, as well as differences in the asymptotes of our sigmoid fits. We quantified differences in lick counts between rightward (magenta) and leftward (cyan) adaption for high-coherence rightward test motion (Fig. 6*C*, inset) and for high-coherence leftward test motion (Fig. 6*E*, inset). In both cases for all four mice, there were no significant differences in lick counts (Fig. 6*C*,*E*, magenta vs cyan data points; *p* > 0.05, bootstrapping). Correspondingly, we tested for differences in the lower (Fig. 6*D*, inset) and upper (Fig. 6*F*, inset) asymptotes of our sigmoid fits for rightward (magenta) and leftward (cyan) adaptation. For all four mice, there were no significant differences in both the lower and upper asymptotes of the sigmoid fits for rightward versus leftward adapting conditions (Fig. 6*D*,*F*; *p* > 0.05, bootstrapping). Overall, our data demonstrate that opposite directions of motion in the adapting stimuli resulted in significant lateral shifts, but not in any significant upward or downward shifts, in how licking behavior changes with motion coherence.

**Figure 7.**
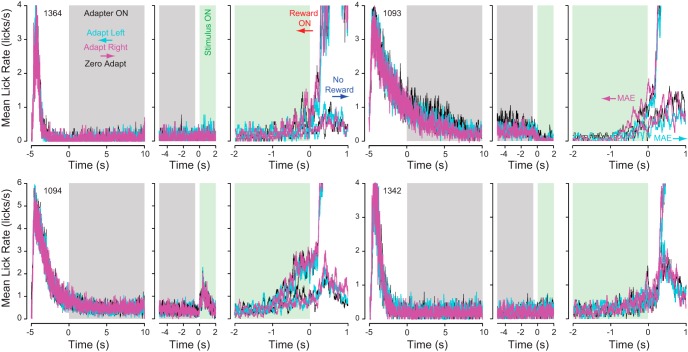
The influence of the adapter stimuli on licking behavior. The average lick rate over the period of the experiment is shown to illustrate differences in lick behavior between the three adapting stimuli (left, right, and 0) during the adapt (left), test (center), and reward (right) phases. Lick rates differ between rightward (magenta) and leftward (cyan) adapting stimuli only before reward onset (right plots, 0 s). There is no change in lick rates versus time at the onset of the adapter stimulus (gray box, left plots, 0 s).

**Figure 8.**
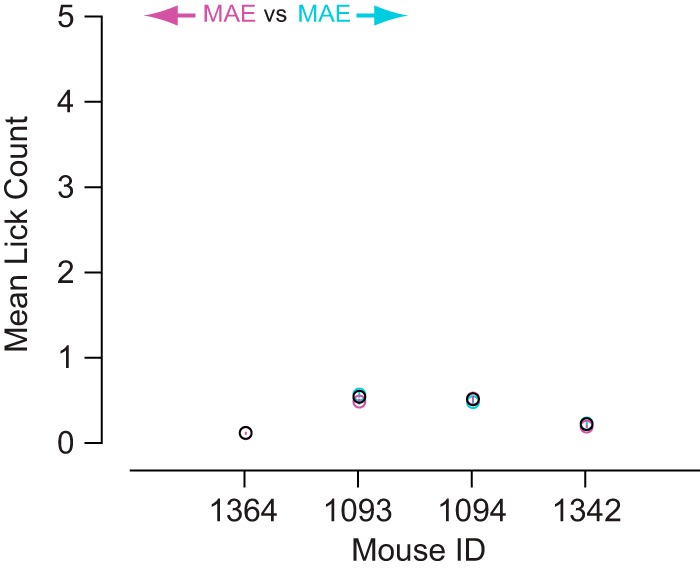
Licking during the adapting phase was infrequent and unrelated to the adapter stimuli. For all four mice, during the adapting phase, average lick rates were ≤0.6 licks/s and were not significantly different between rightward (magenta) and leftward (cyan) adapting conditions (*p* > 0.05). Error bars are SE of the average lick rate and open black circles are the lick counts for the 0% coherence adapting condition (*n* = 806–910 repeats for each condition).

If mice licked more often for leftward versus rightward adaptation during the adapting phase (expecting a reward based on their past conditioning), they might be more fatigued and lick less often during the test phase leading to less licking for leftward (cyan) versus rightward (magenta) adapted behavioral curves. Therefore, to directly determine if mice reacted to the adapting stimuli in our experiments, we investigated lick behavior during the adapting phase. Figure 7 shows how the lick rate varies over the time course of a trial with respect to rightward (magenta) versus leftward (cyan) adaptation. The trial is broken up into three parts: (1) Adapt Phase, the first 10 s of adaptation (random interval of at least 10 s) aligned to the onset of the adapter stimulus (at 5 s after the reward time for previous trials); (2) Test Phase, the final 5 s of adaptation and the first 2 s of the test stimulus aligned to stimulus onset; and (3) Reward Phase, the final 2 s of the test stimulus and 1 s following reward onset (at 0 s). First, the lick rate decays following licking in response to rewards from the previous trial (Fig. 7, peak farthest to the left). Then, there is no apparent change in lick rate with respect to the onset of adapter stimuli during the adapt phase (Fig. 7, gray). The mice only lick spontaneously and infrequently during the adapting phase and then there is either no change in lick behavior (Fig. 7, green, Mice 1364 and 1342), a decrease from spontaneous licking (Fig. 7, green, Mouse 1093), or a transient increase from spontaneous licking at stimulus onset (Fig. 7, green, Mouse 1094). Lastly, licking increased for all four mice preceding reward onset (Fig. 7, right side of green region) and the licking is greater for rightward adaptation (Fig. 7, magenta) compared to leftward adaptation (Fig. 7, cyan). To confirm that there was no direction-dependent reaction to the adapter stimulus, we compared the lick rates between rightward and leftward adaptation and found that licking was infrequent (<0.6 licks/s) and not significantly different between conditions (Fig. 8, magenta vs cyan data points; *p* > 0.05, bootstrapping). These observations suggest that the mice were ignoring the low contrast adapter stimuli.

## Discussion

We have demonstrated that mice can accurately distinguish between leftward and rightward stimuli by using classical conditioning paradigm to associate water reward with motion. While rats and other animals have previously been shown to perform direction discrimination tasks (Douglas et al., 2006; Stirman et al., 2016), early attempts to train mice to perform opposite direction discrimination were not successful (Douglas et al., 2006). This failure could either be ascribed to differences in the neuronal representation for motion or to issues with the training paradigm. We used a two stage protocol to train the animals in which animals initially learned to distinguish horizontal and vertical motion and then a protocol was used in which we rotated, over a period of days, the unrewarded upward motion stimulus until the animals were performing the task using only horizontal motion. Because another recent study used licking without rotating the direction of motion to successfully train mice to discriminate motion in opposite directions (Marques et al., 2018), our use of licking behavior to test for motion discrimination performance may also be an important factor to capture this ability in mice. Once the animals were trained for this task, we were also able to demonstrate that their report is subject to the waterfall illusion: e.g., prolonged visual stimulation with rightward motion induces mice to report that leftward motion exists for conditions in which no coherent motion is presented. Our results indicate that the mouse is able to discriminate leftward from rightward motion and that discrimination is susceptible to recent motion history.

The performance of our mice in discriminating the direction of motion with reduced coherence are similar to what has been observed in previous studies (Douglas et al., 2006; Stirman et al., 2016; Marques et al., 2018). The behavioral shifts when preceding the test motion with an adapter are consistent with the motion aftereffect shifts observed in psychometric functions for humans (Blake and Hiris, 1993; Van Wezel and Britten, 2002; Czuba et al., 2011). Furthermore, our analyses of the behavioral changes induced by the adaptor stimuli demonstrate that state changes such as arousal or lick fatigue cannot account for the shifts in behavior we observe (Figs. 6–8). The magnitude of the shift in sigmoid functions that we measured in mice is smaller than what has been observed in humans, but that can be attributed to differences in durations and contrasts for both the adapting and test stimuli. For example, increasing the contrast of the adapter or decreasing the contrast of the test stimulus increase the strength of a motion aftereffect (Keck et al., 1976).

Motion information emerges at multiple stages in the mouse visual system, which may underlie the discrimination behavior we observe. Mice have direction selective cells in their retina (Elstrott et al., 2008; Yonehara et al., 2009; Cruz-Martín et al., 2014; Hillier et al., 2017; Shi et al., 2017), lateral geniculate nucleus (Marshel et al., 2012; Piscopo et al., 2013; Sun et al., 2016), primary visual cortex (Drager, 1975; Niell and Stryker, 2008; Marshel et al., 2011; Rochefort et al., 2011), and the superior colliculus (Drager and Hubel, 1975; Shi et al., 2017). Many questions still remain about the specific circuitry that produces the motion aftereffect. Barlow and Hill (1963) recorded from retinal ganglion cells in the rabbit and discovered that spontaneous activity was reduced following prolonged exposure to the preferred direction of motion, but there was no change to spontaneous activity following prolonged exposure to the opposite direction of motion. This imbalance of activity following the stimulus would lead to a perception of motion in the opposite direction. The direction selective neurons in mouse retina (Elstrott et al., 2008; Yonehara et al., 2009; Cruz-Martín et al., 2014; Hillier et al., 2017; Shi et al., 2017) may adapt in a similar manner. Recent studies also suggest that direction selectivity independently develops in the visual cortex of mice (Hillier et al., 2017; Lien and Scanziani, 2018; Marques et al., 2018). Similar to the results of Barlow and Hill (1963), experiments in the cat demonstrate that there is reduced activity following the exposure to the preferred direction of motion and no change in activity following the exposure to the opposite direction of motion in cells in the primary visual cortex (Priebe et al., 2010). This simple reduction in activity would not require any special connectivity or organization among direction selective cells and would be a plausible in explaining the motion aftereffect in primates, carnivores, and rodents. Contrast-dependent adaptation properties in the primary visual cortex are similar for mice compared with cats and primates (Ohzawa et al., 1985; Sclar et al., 1989; Bonds, 1991; Crowder et al., 2006, 2008; Kohn, 2007; Stroud et al., 2012).

Recordings in higher areas such as MT in owl monkeys, however, reveal not only a reduction in response following exposure to the preferred direction of motion, but also an enhancement of responses following exposure to the opposite direction of motion, suggesting that competitive circuitry might also contribute to motion processing and the motion aftereffect (Petersen et al., 1985). This was confirmed more recently in area MT of macaque monkeys as well (Kohn and Movshon, 2003). This competition would require specific inhibitory connections between cells with opposing preferred directions of motion or a columnar organization where cells with opposing preferred directions are located next to each other (Malonek et al., 1994; Ohki et al., 2005), which have not been observed in rodents (Ohki et al., 2005). The lack of organization in rodent visual cortex may lead to differences in adaptation properties compared with cats and primates (King and Crowder, 2018), which might be also true for the motion aftereffect.

Recent sensory history is critical to interpreting new incoming sensory information. This is particularly relevant when that incoming information is noisy, uncertain, or ambiguous. The motion aftereffect is an illusion that arises out of the circuitry that helps integrate past and present motion information. Such motion processing is critical to several species, including mice, in stabilizing images while navigating in an environment, avoiding moving objects, or interacting with moving objects and the signatures for this processing appear to be preserved across mammalian species.

## Synthesis

Reviewing Editor: Tatiana Pasternak, University of Rochester Decisions are customarily a result of the Reviewing Editor and the peer reviewers coming together and discussing their recommendations until a consensus is reached. When revisions are invited, a fact-based synthesis statement explaining their decision and outlining what is needed to prepare a revision will be listed below. The following reviewer(s) agreed to reveal their identity: Laura Busse.

The two reviewers found the revised manuscript much improved but raised a number of additional issues that must be addressed before the paper is accepted for publication in eNeuro. These are summarized below.

1. Please perform an additional more rigorous analysis that will deal more adequately with the likely shifts in arousal and criteria. Reviewer 1 suggests to fit sigmoid curves to the data to estimate upper and lower asymptotes that would allow a better assessment of the reasons for changes in the lick rate.

2. Discuss possible behavioral changes associated with adaptation (bias, arousal, task engagement)

3. Please address the question raised by Reviewer 2 concerning 0% coherence condition and individual differences across mice. Can you respond to the possibility raised by this reviewer that the observed effect reflects the change in strategy or fatigue in the adapting phase, rather than sensory adaptation?

4. Please include recent relevant citations mentioned by Reviewer 2.

5. Both reviewers also identified minor problems in the text and in the figures. Please address those.

REVIEWER 1

ADVANCES IN THE FIELD

The work shows a useful behavioral training method, and also describes a motion adaptation effect that is of interest. The core of the paper is the motion adaptation result, which is interesting and useful, and strengthened by added data, but I have a request for additional analysis.

COMMENTS TO THE AUTHORS

This is a major revision of the earlier submission. A good amount of new data has been added, increasing the number of animals in the main adaptation experiment to four, and some parts have been removed, for example the interocular transfer data.

The work shows a useful behavioral training method, and also describes a motion adaptation effect. The core of the paper is the motion adaptation result, which is interesting and useful, and strengthened by added data, but I have a request for additional analysis.

- Major: adaptation experiment

The adaptation experiment is now shown for four animals, and the analysis also has been improved (but some additional analyses are still desirable). The work is done in a detection or yes/no paradigm where spurious effects can be produced by changes in animals' willingness to respond, and to make firm conclusions such effects must be ruled out. Here, the behavioral metric used is mean lick count, and the authors show lick count as a function of varying coherence of the stimulus (Fig. 5). The lick count plots are sigmoidal and largely saturate at high coherence in each direction. The required analysis is not the same as would be used in typical detection task with binary outcomes, but it is possible to use these data to rule out changes in willingness to respond (e.g. arousal or criterion shifts). First, if animals change their overall arousal, this would be seen as a change in lick rate that affects every coherence, offseting the entire curve (i.e. both asymptotes). If animals' motivation or engagement changes, this would be seen as either a slope change or an opposite shift in both asymptotes, where decreases in effort cause both asymptotes to move towards the zero coherence level, and vice versa. And if animals develop a bias toward one direction, this should at least change one asymptote but not the other. (These possible behavioral changes with adaptation (arousal, engagement, bias, etc.) are not well explained in the paper -- the analysis in Fig. 6B appears to come out of nowhere for readers unfamiliar with yes/no task details -- and this explanation must be added in detail in Results, Discussion.)

Fig. 6B is shown as a control for these possible behavioral variations, but more analysis is needed. The authors use the central four coherence levels for their aftereffect analyses (susceptible to cherry-picking/p-hacking concerns). The use mean lick rate (i.e. lower asymptote) as a control for changes in willingness to respond, which is fine, but the other asymptote is not analyzed. These analyses are in the correct spirit, but a more rigorous approach is required, in my view. The authors should fit two sigmoidal curves to the data (Fig. 5B), and estimate for each adaptation diretion (1) coherence threshold, (2) upper asymptote, and (3) lower asymptote or mean lick rate. (It is not necessary to fit a slope for each direction; a single slope would be sufficient). This approach would remove cherry-picking concerns and add abiity to track both asymptotes. These estimates should then be plotted with confidence bounds in the same format as Fig. 6B. It appears from Fig 5B that the asymptotes indeed do not vary with adaptation, and the threshold shifts also appear small but significant, supporting the present conclusion. But this more rigorous approach must be added.

Also, lick rate time window: please add analysis to new panel(s) in Fig. 3: use a window of fixed duration and change time offset while calculating d-prime.

- Minor

+ Fig 2A - dashed line, label in legend?

+ Fig 5: dashed and solid lines, label in leged.

+ lack of redline version makes submission hard to review.

REVIEWER 2

COMMENTS TO THE AUTHORS

This manuscript is a well-done, small behavioral study in mice, showing that mice can be trained to perform a motion discrimination task. In addition, it demonstrates that some features of the behaviour are consistent with the effects predicted from a motion aftereffect.

I have not seen the previous version of the paper, so I have 2 major concerns:

- l. 254-256: "Note that the fits for 0% coherence adaptation from Fig. 4A are replotted in Fig. 5B (black traces) and lie between the leftward and rightward adapting conditions."

I am not convinced by this statement, as some of the psychometric curves after adaptation fall directly onto those obtained with 0% coherent adaptation. Why is this the case? Even more puzzling, why is it different across mice, which of the two adaptation conditions is similar to the 0% coherence condition? Could this indicate that the effect investigated is not so much an adaptation effect, which should happen automatically and consistently, also relative to the 0% coherence, but a signature of a strategy or task confusion or tiredness from licking in the adapting phase?

I see that the authors have included a figure for reviewer 1, who raised similar concerns. Since this is an important issue, please provide more than "an example for one mouse". Please show all the data, and perform the relevant statistics to demonstrate that statistically, there is indeed no difference during adaptation across mice.

- p. 2, l. 40 is not true because there is (now) a published paper demonstrating motion direction discrimination (2AFC) in head-fixed mice

- Marques, T., Summers, M.T., Fioreze, G., Fridman, M., Dias, R.F., Feller, M.B., and Petreanu, L. (2018). A Role for Mouse Primary Visual Cortex in Motion Perception. Current Biology 28, 1703-1713.e6.

MINOR:

- Fig. 2 and 3: I find it very confusing that time is expressed relative to stimulus onset in the figures, whereas in the description in the text, time is relative to reward onset. Please express everything relative to reward onset.

- The end performance of mouse 1364 in Fig. 2B does not look very impressive, which is partly due to the huge decrease of overall licking. Maybe the authors want to consider performing their analysis on an index, which normalises for this difference. See e.g. Jurjut et al. (2017)

- l. 217: What do the authors mean by "even though the task continued to become more difficult." I don't see how difficulty is assessed here, and what the evidence is that opposing motion directions are more difficult for mice (other than some paper with a different method having that finding)

- Fig. 6b: please add mouse labels

- Please consider adding Jurjut, O., Georgieva, P., Busse, L., and Katzner, S. (2017). Learning Enhances Sensory Processing in Mouse V1 before Improving Behavior. J. Neurosci. 37, 6460-6474. as another reference using this paradigm.
